# Gerotranscendence and Alaska Native Successful Aging in the Aleutian Pribilof Islands, Alaska

**DOI:** 10.1007/s10823-020-09421-9

**Published:** 2021-02-10

**Authors:** Erik S. Wortman, Jordan P. Lewis

**Affiliations:** 1 University of Washington School of Medicine, 1959 NE Pacific St, Seattle, WA 98195 USA; 2 University of Minnesota Medical School, 624 E. 1st. Street, Suite 201, Duluth, MN 55805 USA

**Keywords:** Successful aging, Gerotranscendence, Alaska native, Healthy aging, Rural

## Abstract

The population of the United States is aging and by 2045 it is projected that approximately 1 in every 6 Alaskans will be 65+. Delivering healthcare and meeting the needs of older Alaskans in their community is critical to supporting healthy aging and community sustainability. Alaska Native (AN) Elders are underserved with very few studies providing an emic perspective on their experience aging. This research opens the door and allows us a glimpse of the AN Elder experience of aging: the values, beliefs, and behaviors that allow them to age well. This study highlights the characteristics and activities of AN Elders in the Aleutian and Pribilof Islands to further develop the model of AN successful aging. There are many theories of aging and this study explores a cross-cultural understanding of gerotranscendence - the personal and interpersonal changes that result from successful aging or achieving Eldership. This study interviewed Elders in two communities of the Aleutian and Pribilof Islands region. Using 22 standardized questions based on the explanatory model, researchers facilitated discussion of what it means to be an Elder and age successfully. Employing thematic analysis, interview transcripts were analyzed for themes to organize the data. Themes were organized into 5 core elements of successful aging with specific emphasis on values, beliefs, and behaviors that were protective and helped them adapt to aging-related changes. Interview content, meaning, and themes support the four elements of the AN model of successful aging developed by Lewis (*The Gerontologist, 51*(4), 540-549, 2011): *Mental and Emotional Wellbeing*, *Spirituality*, *Purposefulness and Engagement*, and *Physical Health*. Elders’ stories highlight the importance of reflection, personal growth, and psychosocial development. Elders who more strongly identified with their role in the community described how their perspective had changed and they shared stories that emphasized culture, connection to the land, and enjoyment of daily activities that resulted in increased life satisfaction. Elders provided clear evidence that they experienced aspects of gerotranscendence, which Tornstam (*Journal of Aging Studies, 11*(2), 143-154, 1997) categorized as the *cosmic* dimension, the *self*, and *social and personal relationships*. Elders adapting to aging-related changes and embracing their role as an Elder provided the greatest evidence of gerotranscendence - they developed new perspectives on life, took on new roles within the community, and experienced a shift in mindset that reinforced the importance of culture, tradition, and *the Native Way of Life*. This research allowed AN Elders to share their experiences, define successful aging, and expand the concept of Eldership to include changes in mindset, values, and relationships with themselves and others. The study is a framework to help us better understand the experiences of AN Elders aging successfully and the wisdom they wish to impart to others to help them learn to live healthy and meaningful lives.

## Introduction

Historically, the study of successful aging has overlooked culturally-based conceptualizations of aging (Torres 1999) and has failed to account for the multidimensionality of the aging person (Flood 2002). Our understanding of successful aging has continued to change to incorporate other aspects of psychosocial development, including gerotranscendence (Tornstam 2005). Tornstam (1994) defined gerotranscendence as a transition from a materialistic perspective to a more cosmic and transcendent view of life accompanying the process of aging. More simply, older adults experience increased feelings of connection with past generations and a decreased interest in unnecessary social interaction. This transition during the aging process implies a redefinition of time, place, life and death, and a new understanding of the self across three levels, which will be discussed later.

Tornstam’s gerotranscendence theory is considered one of “positive aging” (Tornstam 2005), an antecedent of successful aging and Lewis (2011) found Alaska Native (AN) Elders defined successful aging holistically and gerotranscendence as an important element that supports AN Elders’ aging process. Recent successful aging studies highlight the importance of maintaining optimal health and functional ability (physical and mental) (Gondo et al. 2013), and given this biomedical focus, many older adults from racial and ethnic minority groups do not meet the criteria. This study expands Lewis’s (2011) AN model of successful aging to include gerotranscendence as a theoretic framework to help AN Elders understand the *process* of successful aging and authenticates the characteristics and behaviors of those aging successfully. Creating new understandings and ways to appreciate Elders’ experiences will help inform and educate family, community, health, and social service providers who can positively impact the aging process.

## Historical Context

It is important to understand and be sensitive to the historical context of the Alaska Native community where Elders have lived and the impact of historical events on their psychosocial development. The text, “Slaves of the Harvest,” written by Torrey and Krukoff (1978) provides a comprehensive account of historical events including the first contact with outsiders, exposure to foreign disease, missionization, colonization, and oppression, as well as survivance (combining survival and resistance) to harness and emphasize indigenous people’s strength and thriving (Vizenor 2008).

Within the context of this study, many AN Elders recalled stories from their parents about the WWII internment experience and the governmental regime under which they used to live. Memories of people who never returned, extremely harsh living conditions, and the loss of whole village sites are trigger points for psychosocial pain and trauma. When the traumatic response of the body - the fight, flight, or freeze response - becomes embedded it fundamentally changes our emotional interaction with the world around us. The term *historical trauma* is used to describe the constellation of symptoms that occur as a result of cumulative emotional and psychological trauma (Yellow Horse Brave Heart 2003), which can be transmitted across the life span or generations (Denham 2008). Research in psychobiology informs us of the connection between the deep emotional response to trauma and the somatic response conveyed to the body (Conching and Thayer 2019), referred to as epigenetic modifications (Thayer and Non 2015). Epigenetic modifications cause changes in gene expression that can impact physiological systems (McDade et al. 2017; Thayer and Non 2015), including stress, immune, and cardiovascular systems, resulting in increased chronic disease risk, even across generations (Conching and Thayer 2019). The embodiment of trauma and the way it is transmitted interpersonally and between generations has an impact on Elders’ lives, their health, as well as the well-being of their families and communities. For example, AN Elders may not remember traditional dances from their Elders and is unable to teach the youth this once important cultural expression and celebration. This lack of knowledge is traced back to the missionaries banning traditional dancing because their beliefs it was evil, eliminating dancing from their lives completely. This stress and feelings of guilt and shame about dancing may manifest itself as depression and maladaptive behaviors, which are passed down to their children and grandchildren. The Elders in this study emphasized the importance of never forgetting this history and to pass those memories down to future generations as a means of metabolizing this trauma, healing, and strengthening future generations. This aspect of sharing and teaching the youth is a way of healing all generations and an integral part of healthy aging across many racial and ethnic minority groups, especially for Indigenous populations (Lewis and Allen 2017).

## Theories of Aging

Within the successful aging literature, Tornstam (1997) proposed gerotranscendence as a developmental step, in which a person shifts their “perspective from a materialistic and pragmatic view of the world to a more transcendent one, normally accompanied by an increase in life satisfaction” (p. 143). The theory of gerotranscendence is based on psychosocial development theory and states that human development is a process continuing into old age, and this process, when realized fully, ends in a new perspective on life and aging (Wadensten and Carlsson 2003). During this transformative process, an older adult is motivated to resolve and overcome past challenges, while coping with the physical changes that happen before death, resulting in a redefinition of self. As Tornstam (2011) stated, “As we age and mature, we learn to handle life better” (pg. 167).

Tornstam and others proposed this shift in perspective occurs in three dimensions: the *cosmic*, the *self,* and *social and personal relationships* (Rajani and Jawaid 2015). The *cosmic* dimension focuses on existential changes (Tornstam 2011) and older adults see themselves connected to something larger than themselves (Buchanan et al. 2016). They experience a stronger connection to past and future generations, changed perceptions of life and death, and gain a new perspective on life and what they have experienced. The dimension of *self* refers to older adults’ changed view of self; they become less self-centered and have new appreciations for life, discover new aspects of their lives, fear death less, and accept the life transitions leading to death (Tornstam 2011). The dimension of *social and personal relationships* highlights the transitions older adults make as they break away from expected roles in family and community. They may become less judgmental and react with an open mind, which may be new behaviors. Older adults may also decrease their number of relationships to focus on more meaningful associations (Tornstam 2011). These three dimensions of gerotranscendence build upon the major aging theories of activity theory and disengagement theory (see Settersen and Godlewski 2016 for an overview of these aging theories).

Activity theory (Havighurst and Albrecht 1953) posits that maintaining the same level of activity from middle adulthood into old age results in life satisfaction for older adults (Lemon et al. 1972). In contrast, disengagement theory states that older adults begin to withdraw from activity, roles, and society as a whole as they transition from middle to older adulthood (Cumming et al. 1960; Richardson and Barusch 2006; Wadensten 2006). Tornstam’s theory of gerotranscendence countered activity theory by asserting that disengagement was a natural part of the human development process, and that time away from activity - spent in isolation and used for reflection and review - was critical to achieving personal growth toward the end of life.

Guided by the Disengagement Theory, Tornstam noted that although older adults tended to withdraw, they were experiencing a reorientation to a more reflective and satisfying state of being and thinking (Dalby 2006; Jönson and Magnusson 2001; Tornstam 2011), contributing to their development of gerotranscendence. Tornstam (1999) also found older adults with higher degrees of gerotranscendence reported higher levels of ability to control their own physical and social activities, were more satisfied with their adjusted social activity, and reported higher life satisfaction. Tornstam, however, suggested that gerotranscendence cannot be viewed simplistically as a new form of social withdrawal, rather, gerotranscendence moves “beyond the dualism of activity and disengagement” to present a new process whereby people redefine themselves outside of typically accepted labels and cognitive boundaries (Tornstam 2011, p.166).

Erikson’s (1963) last stage of psychosocial development, Ego Integrity, refers to an individual’s acceptance of his or her own life and being able to integrate the elements of their lives that have passed (Tornstam 2011), how they fit together to make a whole (Wadensten 2005) and provide them with a sense of meaning. Leading gerontologists (Butler 1963; Birren 1964) have explored the role of personal meaning and believing that one’s life is worthwhile and significant in the development task of achieving ego integrity (Erikson 1963). An individual who has achieved ego-integrity is described as “having a balanced investment in self as well as in others, and as having moved from concerns with things to ideas, from actions to meanings” (Birren and Renner 1980, p. 28), and those who do not achieve this stage experience despair (Erikson 1963).

There is no conclusive evidence that gerotranscendence is universal to all cultures, or religious backgrounds, however, this study provides strong initial evidence supporting AN Elders’ experiences with gerotranscendence. Eliciting AN Elders’ perspectives allows them an opportunity to define what it means for them to age successfully and explain their continued psychosocial development.

## Design and Methods

This study is a subset of the parent study, funded by the National Science Foundation, that explored AN successful aging in two rural regions of Alaska (Aleutian Pribilof Islands, Norton Sound Southern Sub-region) and the urban center of Southcentral Alaska (Anchorage and the Mat-Su Valley). For this study we used a nominative sample: AN Elders were nominated by their community and tribal councils as community members they considered to be successfully aging or achieving Eldership, and fulfilling the roles and duties expected of AN Elders (e.g., role models, mentors, leaders, and so on).

Semi-structured, in-depth, interviews were conducted in the community, one-on-one in the Elder’s homes, community center, or other location of their choosing using 22 standardized questions. Questions were a mix of open-ended and closed questions based on the Explanatory Model Interview Protocol (Kleinman 1980) covering topics such as how AN Elders define successful aging, what helps them to age well, as well as whether or not they believe their community is supportive of them aging successfully. Questions also asked what it means to age poorly and how they avoid poor aging. Follow up questions were asked to clarify and follow up on details. Interview questions can be found in Appendix A. Elders were allowed to ask questions of the interviewer, provide feedback about the interview process, and able to stop the interview at any point for any reason. An interpreter was made available for any Elder that wanted to participate in their native language. The interview was recorded and transcribed for analysis. (See Lewis 2011 for more on parent study methods.)

### Demographics

Twenty-nine AN Elders from two rural communities were interviewed. Twenty AN Elders currently living in one of the two rural communities and nine born in the communities but currently living in Anchorage and the Matanuska Susitna Valley. Participants from the two rural communities were Unalaska (11) and St. George (9). The age range was 46 and 87, with an average age of 67 (Table 1). Collectively the rural participants had 1251 years of lived experience in the region. Most participants were women (19), married (12), and were at least high school graduates (23). Thirteen Elders indicated having learned the Aleut/Unangax language as their first language.

**Table 1:** Participant demographics

Total		Average Age	First Language - (English), [Aleut], {Other}	Marital Status - (single), [married or cohabitate], {divorced}, |widowed|	Education - (less than high school), [high school grad or equivalent], {some college}, |college degree or graduate|
APIA Villages					
*Male*	7	69	(4), [3], {0}	(2), [3], {1}, |1|	(2), [2], {3}, |0|
*Female*	13	70	(6), [6], {1}	(1), [3], {5}, |4|	(4), [6], {3}, |0|
APIA Urban-Southcentral					
*Male*	3	67	(3), [0], {0}	(0), [2], {0}, |1|	(0), [3], {0}, |0|
*Female*	6	60	(2), [4], {0}	(1), [4], {1}, |0|	(0), [2], {2}, |2|

### Data Analysis

The qualitative data provided information on successful aging in rural Alaska, what aging means to Elders, and what needs must be met to ensure they age successfully in their community. An inductive research strategy was used in which ideas, concepts, and themes emerged from the data by beginning an inquiry without a priori definitions or hypotheses about what will be discovered (Yegidis et al. 1999).

**Fig. 1:**
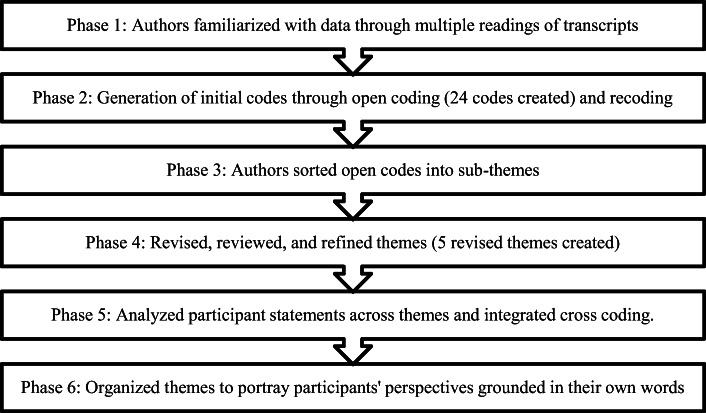
Data Analysis Flow Chart

Thematic analysis (Braun and Clarke 2012; Fereday and Muir-Cochrane 2006; Johnson & Onwuegbuzie et al. 2009) was performed through the process of open and axial coding to create meaningful patterns related to successful aging (see Fig. 1 for data analysis flowchart). In open coding, the two authors became familiar with the data by reading the interview transcripts multiple times and keeping notes. Latent codes, or the unobservable constructs, were created to capture participants’ experiences of successful aging based on the interpretive analysis of the authors (Clarke et al. 2015; Neuendorf 2019) and 24 initial codes were developed to organize the data into a theoretical fashion relevant to the study. Next, the authors organized the codes into thematic clusters, referred to as axial coding, which served as the larger themes used to explore the process of successful aging. A sample of representative quotes and coding is included in Appendix 2 Table 6. Once the codes were clustered, both authors met to discuss the cross-coding, modify the themes (Boyatzis 1998) and identified broader patterns of meaning (see Table 2). The two authors explored how the themes related to one another and if they told a convincing story that illuminated the experiences and processes of successful aging (Neuendorf 2019; Nowell et al. 2017). Next, we used selective coding to establish the five core elements, or themes (Corbin and Strauss 1990) of successful aging for AN Elders and the authors wrote up the findings (Alholjailan 2012). Adhering to principles of CBPR, which are particularly well-suited for research with tribal communities (Rasmus 2014), the authors presented preliminary findings to participating communities to solicit feedback and ensure analyses and findings were representative of the Elders’ experiences and understandings of successful aging (Lewis 2011).

**Table 2:** Codes grouped into themes from inductive thematic analysis, (# of interviews) [# of times coded]

Theme	Physical Health and Mobility	Emotional Wellbeing	Purposefulness and Engagement	Spirituality	Gerotranscendence
Codes	Community resources (19) [154]	Education (22) [117]	APIA (16) [42]	Religion/spirituality (19) [103]	Advice for youth (16) [57]
	Physical Health (25) [389]	Emotional well-being (25) [391]	Community engagement (25) [497]	Native way of life (24) [395]	Alcohol and drugs (21) [72]
	Stay active (24) [173]	Family (25) [599]	Housing (11) [24]		Becoming an Elder (24) [115]
	Transportation (14) [48]	Laughter (17) [226]	Role of Elder (16) [54]		Change (25) [195]
		Rural vs Urban aging (24) [188]	Work (22) [181]		History (12) [44]
		Subsistence (20) [160]			Knowing how to age (23) [73]
					Technology (16) [34]

## Results

This study looked at the concept of successful aging or Eldership and how AN Elders’ perspectives change as they more fully recognize and embrace their role as an Elder. The findings supported the four elements of the AN model of successful aging developed by Lewis (2011): *Mental and Emotional Wellbeing*, *Spirituality*, *Purposefulness and Engagement*, and *Physical Health*, and though the experiences of Elders were unique to the region they are thematically consistent with previous research on the subject (Lewis 2011, 2013a, b, 2014a, b).

Elder participants shared stories of the past including important events in their lives and the lives of earlier generations highlighting the importance of reflection, personal growth, and psychosocial development. Elders who more strongly identified with their role in the community described how their perspective had changed and they shared stories that emphasized culture, connection to the land, and enjoyment of daily activities that resulted in increased life satisfaction.

The Elders in this study also provided clear evidence that they experienced aspects of gerotranscendence, which builds upon the earlier work developed by Lewis. Tornstam (1997) described and categorized the qualitative changes Elders undergo as they experience gerotranscendence, which include the *cosmic* dimension, the *self*, and *social and personal relationships*. The following section will outline Tornstam’s categories and provide the Alaska Native Elders’ context with a quote to illustrate the meaning in their own words.

### Cosmic Dimension

Tornstam (2011) explained the cosmic dimension as a reinterpretation of past experiences in which “the distance between the past and present is disappearing” (pg. 169). Elders described the importance of participating in traditional subsistence activities for personal health (physical, mental, spiritual) and maintaining a connection to ancestors through cultural activities (e.g., dancing, singing, arts and crafts, subsistence harvesting and gathering). The following table illustrates the *Comic Dimension* (Table 3).

**Table 3:** The Cosmic Dimension

The Cosmic Dimension (Tornstam 1997)	Alaska Native Successful Aging	Quote
Time and Childhood		
Reinterpretation and reconciliation of past events with boundaries of time less important.	Sharing stories of the past and childhood; including hardship and adversity that give meaning and purpose.	“*I think passing on the good things that you’ve learned from bad things that have happened to you in your family and resolving things that have been hurting families or causing families to break up*.”
Connection to Earlier Generations		
Feeling of connection with former, present and future generations.	Connecting with youth affirms their religion, culture, tradition and “native way of life.”	“*Once the Elders are gone, how are you going to learn all this stuff? You sure in heck ain’t going to the library to look at Ray Hudson’s book or somebody that wrote books on the Native life*.”
Life and Death		
Heightened enjoyment of life with fear of death lessened.	Desire to spend time and remaining years in community.	“*But I love it here. I was born and raised here. I like the quietness. I love the clean air and I love the ocean. I love the birds and stuff. And I have my books I, I mean, I read. I would read all of the time. I would read probably about five to six books a week*.”
Mystery of Life		
Acceptance of unknown and limits of understanding.	Focus on knowledge gained through lived experience and traditional understanding.	“*That’s how we learned, we learned from other people, from our own experiences. Our own experiences would probably be trial and error but we could learn a lot by paying attention to what other people try to tell us. So, learn to do many different things, not just do one single job*.”
Rejoicing		
Increasing enjoyment in everyday events.	Appreciation of daily activities and visiting with friends and family.	“*Marge comes over all the time. Sassy will visit. Nina always talks on the phone. My sister calls almost every day. She lives in Chicago*.”

Tornstam (2011) also highlighted how Elders developed a strong connection to previous generations, creating a “link in the generational chain, where the chain itself is important, not me” (pg. 169). This was reiterated frequently in many interviews as a strong desire to pass on *the Native way of life*.

Gerotranscendence helps us understand how Elders come to reconcile life and death, being thankful and appreciative of life and also not be overly attached to material items. Several Elders expressed gratitude when they woke up each day and acknowledge that one day they may not rise with the sun, a reality they have accepted. Elders in the study spoke from personal experience and focused on knowledge gained through lived experience and traditional understanding. Elders’ thoughts and opinions on topics were less hardened and significant the farther conversation extended beyond family, community, place and tradition. Tornstam highlighted that as we grow older we recognize that human intellect may have its limits. Elders shared the joy and happiness they got from deep connection with their community manifest in everyday actions like visiting with family and friends.

### The Self

Many Elders spent time reflecting on their past experiences, sharing stories of poor choices and mistakes they made, which signifies their ability to confront their former self and highlight the positive outcomes and personal benefits of this self-reflection. Tornstam (2011) described this reflective process in which Elders look back at earlier phases in their life and discover hidden meaning in the choices made – the good and bad aspects of a life lived. The following table, *The Self* (Table 4), illustrates how Tornstam and Alaska Native Elders actualize the *Self*

**Table 4:** The Self

The Self (Tornstam 1997)	Alaska Native Successful Aging	Quote
Self-Confrontation		
Discover hidden aspects of self.	The process of self-reflection and reexamining both the good and bad, acknowledging changes.	“*But I think the biggest for me is, I can’t stress it enough, the opportunity to take care of my parents. I am getting so much from them and at times it is stressful but then again, it’s what I was taught to do*.”
Decrease in Self-Centeredness		
Develop new awareness that he or she is not the center of universe.	Taking oneself less seriously, light-heartedness and accepting assistance, all while being resilient.	*“Keep sharing, keep giving back, just information and what you’ve learned, eat well, don’t eat too much, pray a lot, do a lot of forgiveness, forgive everybody, sing in the shower. All those things, look in the mirror and laugh at yourself, laugh a lot. That’s my advice I guess.”*
Body Transcendence		
How to care for body without being obsessed.	Acceptance of the body and physical limitations.	“*You can’t do as much as you used to do. I don’t go out fishing, but my grandchildren do the fishing now*.”
Self-Transcendence		
Shift focus on need of others.	Generativity and engaging with community and family to support others.	“[Mother] *was always helpful, always kind to everybody, always remembering their birthdays and liked to give people gifts, even strangers, welcoming them and being tolerant of even what some people would call the lowest of people. Being kind to them and having an ear for them*.”
Ego-Integrity		
Acceptance of one’s life; pieces of life come together to form a whole.	Developing a coherent story of life, past and present to share knowledge and experience.	*“They don’t know about, like my generation and what we grew up with, how much of a hard time we had, and stuff like that. They don’t understand that. They don’t know it. They’ve never been, seen it, never been through it. So, it’s hard for them to understand what we went through*.”

.

The process of personal growth following self-reflection is well documented in other research by Lewis and Allen (2017) in which Elders spoke about issues of drug and alcohol use and the use of redemptive narratives and generative behaviors and actions. Elders spoke openly and candidly about decisions and behaviors that prevented them from being involved in family, community, and traditional activities and they shared how these behaviors were incompatible with the roles and expectations of being an Elder. By reconnecting with cultural values and beliefs, Elders found reason and motivation for sobriety. The participants in this study highlighted what Tornstam identified as Elders arriving at a middle ground; they are taking themselves less seriously, focusing time on what they deem to be important, and being more selective in their choice of social activities. In addition, they realized that past decisions did not define them, and their past experiences can have a positive impact on others in their family and community.

Tornstam describes body-transcendence as acceptance of the body, appearance, and physical limitations. Several participants considered that an individual becomes an Elder when their physical health declines, they lose mobility, and can no longer do the activities they once could. In small communities, this increases the dependence on family, peers, and local services, which adds depth to social interactions and provides the opportunity for Elders to realize their shifting role as teacher and purveyor of knowledge.

Elders in this study discussed the importance of sharing experiences and traditional knowledge with others. Elders in these communities realized that when their Elders passed on they were responsible for passing on this accumulated wisdom and for many this was a new role, which resulted in a redefinition of self.

Tornstam’s theory of Gerotranscendence borrows the concept of Ego Integrity from Erikson and describes a change in perspective where Elders can integrate different pieces of their life and the experience accrued, where the good and bad became a coherent whole. Acknowledging and integrating personal challenges, adversity, and stories of resilience are integral to the process of self-reflection and illustrates the way Elders process and their perspective evolves. Through this reflection of past experiences and how Elders were able to overcome challenges, they developed a reliable and valuable sense of self.

### Social and Personal Relationships

Elders prioritized whom they spent their time with and what they spent their time doing. For some Elders in this study, there was an increased emphasis on community, cultural activities, caring for grandchildren, and visiting with other Elders in their community. For others, there was an increased need for time to engage in self-reflection and solitude. Many Elders spoke fondly of routine activities they enjoyed, including listening to the radio, doing word puzzles, and cleaning the house. These ordinary and mundane activities allowed time for personal reflection and highlighted a shift in their priorities to emphasize quality personal interaction over quantity. Elders also highlighted the importance of a better relationship with themself and an interest in doing activities alone that others, who are preoccupied, may fail to appreciate. The following table illustrates *Social and Personal Relationships* (Table 5)

**Table 5:** Social and Personal Relationships

Social & Personal Relationships (Tornstam 1997)	Alaska Native Successful Aging	Quote
Changed meaning & Importance of Relations		
Become more selective in their choice of people they spend time with.	Elders prioritized who they spend their time with and what they spend their time doing.	*“You can’t do as much as you used to do. I don’t go out fishing, but my grandchildren do the fishing now.”*
Dealing with Role Playing in Life		
Understand the difference between self and roles played in life; abandon or transcend roles to become closer to genuine self.	Eldership provides opportunity to abandon or transcend roles and redefine important social relationships.	“*Being there for the community when the community needs you for questions or traditional events in the community or questions that the community may have, how things were done to show how the young children, what it is as an elder and to teach their kids to be respectful so they can teach their kids to be respectful towards elders. Yeah, I think more them just being out there just showing that they are respecting the community as the people respect them*.”
Emancipated Innocence		
Develop skills to transcend needless conversations, norms, and rules that curtailed freedom to express self.	The Elders were challenged by aging-related physical ailments and ailments due to family and community roles. Eldership provides opportunity to abandon or transcend roles and redefine important social relationships.	“*Maybe someone will tell it to some other Native. When they’ve finally got their stuff together, got sober and stuff, maybe they’ll tell them about it. So, they’ll hear it and maybe a flicker of light will – you know, maybe they’ll want to learn*.”
Modern Asceticism		
New understanding that last part of life’s journey is easier and more joyful with light luggage; one has enough to meet modern definition life’s necessities and no more.	Elders shared concerns about the younger generation’s preoccupation with technology and popular culture and focus on materialism.	“*Maybe someone will tell it to some other Native. When they’ve finally got their stuff together, got sober and stuff, maybe they’ll tell them about it. So, they’ll hear it and maybe a flicker of light will – you know, maybe they’ll want to learn*.”
Transcendent Everyday Wisdom		
Understanding that finding answers is seldom easy; reluctance to superficially separate right from wrong; discerning when to withhold judgments and advice.	Elders questioned their judgments about the youth being aloof, disinterested and not interacting with Elders; Elders recognize generational differences in understanding the world and reassess criticisms.	“*And I found that to be very sad and I found it true of myself, that I wasn’t looking at teenagers and actually acknowledging them and talking to them. I was guilty of that and so I tried to be more - say hi to them and say their name when I ask them how they are doing*.”

.

The Elders in this study were challenged by physical ailments from an aging body and also ailments due to roles played within their family and community. Eldership provides the opportunity to abandon or transcend roles and redefine important social relationships. For some Elders, this involved acknowledging limitations and giving up their role as a provider. For others, this involved taking on a role as a purveyor of traditional knowledge and culture.

Other Elders described stepping into new roles intentionally to share knowledge from their learned experiences. An Elder spoke about a personal decision to maintain sobriety and a need for others in that situation to step forward and take on a new role as a community leader.

The Elders in this study reported that they were less afraid of judgment from others and did not continue to feel pressure to conform. Elders shared that many social norms were no longer as important to them as they were in the past. Tornstam calls this emancipated innocence and we experienced this when Elders were goofy, telling silly jokes, and playing along with young kids.

Many Elders shared concerns about the younger generation’s preoccupation with technology and popular culture, which is an example of what Tornstam described as modern asceticism. Elders often described kids as captured in a materialistic conception of the world, spending their time on electronic devices and superficial relationships and things (Tornstam 1989). The Elders in this study felt that pop culture materialism will prevent the youth from growing up as a healthy Native person or age successfully.

One Elder described how she questioned her judgments about the younger generation being aloof, disinterested, and not interacting with Elders. She chose to reassess what she was sure of and recognized that rather than the younger generation doing it wrong, she needed to withhold her judgment and, as Tornstam states, transcend everyday wisdom.

## Discussion

Most Elders in this study highlighted continued advancement through life stages or psychosocial development (Erikson and Erikson 1998), which contributed to their understanding of successful aging. In the 8th stage of Erikson’s Stages of Psychosocial Development, *Ego Integrity* versus *Despair*, individuals re-examine their life and are confronted with their evaluation of whether they are content with a happy, productive life or hold onto disappointments and unachieved goals. Erikson’s wife and co-contributor, Joan Erikson, proposed an additional 9th stage of development, described as a psychosocial crisis in which all previous eight stages are encountered again. The central problem, or conflict, she proposed is the attention demanded to one’s loss of capacities and disintegration.

One answer to the conflict raised by Joan Erikson, and well documented in the literature of successful aging, is the theory of gerotranscendence. Gero refers to “old” and transcendence means “rising above;” the process in which Elders recreate themselves with wisdom accrued through life and experience an increase in life satisfaction (Tornstam 1989). This theory explains the developmental shift people experience in old age and helps community members better understand the situational shifts Elders experience in their daily lives. This shift is made from the middle-aged person’s definition of reality based on a materialistic and rational vision to the aging person’s more cosmic and transcendent vision (Tornstam 1992).

Those Elders adapting to aging-related changes and embracing their role as an Elder provided the greatest evidence of achieving gerotranscendence; they developed new perspectives on life, took on new roles within the community, and experienced a shift in mindset that reinforced the importance of culture, tradition, and *the Native Way of Life*. Through achieving gerotranscendence, they have also acquired wisdom, all of which contribute to a higher quality of life and ability to age successfully.

It is important to acknowledge that not all AN Elders are at the same place in their psychosocial development. Tornstam (1997) highlighted that the developmental process towards gerotranscendence can be obstructed or accelerated by life crises and grief, and elements in the culture and environment can also facilitate or impede the process. Gerotranscendence is also not a linear process that individuals progress through, nor is it something worked for, something earned, or what an Elder strives to achieve. Instead, this study taught us how Elders’ perspectives change as a result of accumulated life experiences, interactions with their local environment, culture, and social circumstances; all of which enabled them to adapt and change to age successfully and embrace Eldership.

## Conclusion

Traditionally, healthcare providers have focused on prolonging life, which has been falsely equated with increasing quality of life. In traditional western cultures, people aim to live longer, maintain their independence, and engage in social activities, all of which are championed as evidence of successful aging (Rowe and Kahn 1997). On the other hand, dependence on family or care providers, reliance on community-based social services, and time spent for personal reflection are seen as pathological and not indicators of successful aging. This study reminds us that we must treat the entire person, including their physical ailments and their continued psychosocial development, both of which are integral to successful aging.

This study challenges the Western, medical model of aging and disease where behaviors associated with gerotranscendence are pathological (Buchanan et al. 2015). Rather, this study reminds us to recognize that the behaviors, mindset, and changes AN Elders experience are unique to the culture and context of their region and community. This contextual understanding is important to consider if we wish to support Elders in their later stages of life. This study helps us to understand that as Elders age they can develop new perspectives on life, which in addition to coping with physical ailments, also provides them with increased life satisfaction.

Many Elders expressed concern for the future and *the Native Way of Life*, which exemplifies what Tornstam defined as a shift from a materialistic view to a more transcendent and universal view. For example, the communities in this region of Alaska are experiencing an aging demographic more pronounced than other regions of Alaska. In contrast to many western societies where the changing demographics are often attributed to declining birth rates, in these small island communities there has been an exodus of younger generations for education and employment. Several Elders shared that the absence of a younger generation gave them concern for the future of the community. For some, the lack of interaction with the younger generation and their inability to participate in generative acts has prohibited them from fully actualizing their role as an Elder in their community. Notably absent from the Elders’ interviews was the expressed concern for status, high paying jobs, and monetary wealth. When Elders’ reflected on what was important and identified as a link in the multigenerational changes they were witnessing, they prioritized family, cultural traditions and values, and subsistence activities; the things that define them as Alaska Native and will support the survivance of indigenous culture and *the Native Way of Life*.

We have learned from this study that gerotranscendence is a cross-cultural concept, a later stage in a natural progression toward wisdom and maturation (Rajani and Jawaid 2015). Despite experiencing past hardships and facing limitations due to growing older, AN Elders are thriving, embracing the aging process on their terms, and willing to share experiences that will benefit us all in the future.

## Recommendations

This research reinforces the importance of storytelling and the role of narrative medicine in helping us understand the relational and psychological dimensions of normal or usual aging. The use of the explanatory method to understand patient ideas, beliefs, and values about aging is at the heart of a strengths-based approach to health, wellness, and successful aging. This study reminds us to treat the entire person and employ humility as we strive to understand Elder behaviors, mindset, values, and beliefs within their unique cultural context and life stage. Chimamanda Adichie warns us of “the danger of a single story” and the importance of taking time to understand the individual, recognizing that communities have their own unique cultural context and within the community, there are individuals at varying life stages more or less engaged in personal reflection with different roles in their family, social circles, and community. Importantly, we must continue to build a cross-cultural understanding of gerotranscendence and begin to appreciate the nuanced differences that are rooted in culture, community, and geography.

Person-centered care is grounded in narrative medicine and recognizes the accumulated knowledge of the person and their agency to participate in their treatment; therein the concept of Gerotranscendence takes center stage. Person-centered care includes treatment of specific deficits but more importantly, through the patient’s active engagement in the process, supports and facilitates continued psychosocial development. General guidelines for interventions that support gerotranscendence have been developed (Wadensten and Carlsson 2003) and outcomes studied (Wang et al. 2011) but research is needed to validate the practice and implement them with the support of AN Elders and community organizations.

Healthcare providers are acutely aware that depression, isolation, and loneliness can have a negative impact on Elders’ health and successful aging. According to Tornstam (1997), negative events, or crises, can accelerate the development of gerotranscendence (Read et al. 2014), but little research has explored this relationship closer. Previous studies have found that negative life experiences later in life result in an increased cosmic transcendence (Read et al. 2014), and multiple life crises can accelerate its development (Tornstam 2003). Tornstam (1999) found that those who feared death showed no signs of gerotranscendence, but older adults who were able to shift their perspectives from fearing death to accepting it as a natural part of aging (Tornstam 1997) showed more signs of gerotranscendence. For AN Elders, many have experienced the death of loved one(s) as a result of accidents, drugs and alcohol, suicide, or other traumatic events, and despite their loss, they accept death as a part of life and continue to move forward in life to serve as role models and support their family and community. Based on Tornstam’s theory of gerotranscendence, these traumatic events throughout their life contributes to their development of gerotranscendence. In addition to this relationship with previous personal crises, gerotranscendence is also a new lens to view adaptive behaviors and coping strategies that previously may have been misunderstood as pathological and detrimental to health and successful aging.

Acknowledging gerotranscendence as a process in which Elders continue psychosocial development is significant and provides a framework to understand the changes in behavior, perspective, and social relationships that may outwardly appear maladaptive. Elders describe behaviors unique to their community, culture, and social context and highlight areas of personal growth that allows for increased life satisfaction despite surmounting challenges associated with aging,

Engaging AN Elders to share their experiences helps communities and researchers understand indigenous perspectives on successful aging, specifically what Elders value and what enables them to age successfully. Supporting gerotranscendence may help older adults cultivate positive views of aging, which may lead to improved psychological and spiritual wellbeing (Gondo et al. 2013), improve the lives of AN Elders, and that of the future generations who see them as role models for successful aging. Continued progress will likely have a positive influence on Elder psychosocial development and health outcomes. We would like to dedicate this research and honor the life and work of the late Dr. Howard Luke (1923–2019) of Nenana and Fairbanks, Alaska.

## Appendix 1 Explanatory Model Successful Aging Questionnaire

1. How did you become an Elder?
2. How does it feel to be seen as an Elder in your community?
3. What do you think aging well/good means? What does it mean to you?
4. How did you learn about aging well? From whom? Can you share an example?
5. What is your day to day life like? Can you share an examples?
6. Has aging changed your relationships? With Family? With Friends? With Community? Can you share examples?
7. What helps you age well? How are you able to age well?
8. Why do you think some Elders age well and some do not?
9. How can you tell that an Elder is aging well? Can you share an example?
10. How can you tell that an Elder is aging poorly?
11. What does a person need to do to age well?
12. What does it mean to be an Elder? What is an Elders role?
13. How do you know if someone is an Elder or not? Is it age or something else?
14. Do you think Elders in your community are aging well?
15. Is it different to age today than it was 20 years ago?
16. Who do you think some Elders move away?
17. How do you think aging is different here compared to a city like Anchorage?
18. Do you have any advice for people in your community who want to age well? Or what would you tell them to do to age well?
19. How do you feel about sharing what you know with the youth? What are the benefits? Challenges?
20. Why do you share your experiences with youth? What motivates you?
21. What is the most important thing you want to share with the youth?
22. Is there anything about aging or being an Elder that you want to tell me that I haven’t asked about yet?

## Appendix 2

**Table 6:** Representative quotes from each code with cross-codes

Code	Number of Interviews	Number of Occurrences	Sample/Representative Quote	Additional Codes
Advice for youth	16	57	Keep sharing, keep giving back, just information and what you’ve learned, eat well, don’t eat too much, pray a lot, do a lot of forgiveness, forgive everybody, sing in the shower. All those things, look in the mirror and laugh at yourself, laugh a lot. That’s my advice I guess. - Elder, Unalaska	Emotional well-being, Native way of life, Physical health, Role of Elder
Alcohol and drugs	21	72	My husband and I, of course, we quit drinking six years ago, so we have more respect from people and because our life is around our grandbabies was the reason we quit. We don’t want them growing up in that environment. Their parents don’t drink, which is really good. We stopped that cycle and having that and then plus playing a role in the community where I served on our corporation board, people show that I was boisterous to where I wasn’t before. - Elder APIA in ANC	Family, Community engagement
Aleutian and Pribilof Island Association (APIA)	16	42	APIA is number one. Seeing that really helps, you know, health fairs, and checkups and all that, you know? The health department at APIA is really good. They keep close with [health aids], and there’s all kind of activities, and there’s stuff that people come – just like you guys – come up here and check on the elders and stuff. Yeah, it’s good. - Elder, St. George	Community resources, Emotional well-being, Physical health
Becoming an Elder	24	115	I don’t consider myself an elder yet, but I notice I’ve slowed down since last year. It’s challenging, but I can’t do as much things as I used to do. My plan was, I wanted to share everything I knew with my granddaughters, who are 10 and 8 here in Unalaska. But we always say, we have to wait for snow first. - Elder, Unalaska	Family, Physical health, Native way of life
Change	25	195	To age in a good way is to both physically and mentally work on yourself to process the past to let it go. Hanging onto the past can cause both physical and mental challenges when you get to that senior age, as they call it – the titles they give you, the titles that are used today. So, it is important for one to be able to communicate with if not family members or people they can trust to talk about their post-traumas and finding those ways to process it. That’s the key to being able to enjoy the moment today at your elder age.	Family, Emotional well-being, Community engagement, Physical health
Community engagement	25	497	But then, my days are good because I work on the monthly schedule for our church down there with these materials that we need to read and sing. I enjoy that. That’s what I enjoy doing. - Elder, Unalaska	Emotional well-being, Religion/spirituality, Work
Community resources	19	154	You can’t forget the VPSO, the new one and that other one, Mike. They’re very helpful. Not for violence or anything, but they come if you need to have something. Or he’ll go down to that spice store and he’ll ride them up. So both Mike and Joe have been very, very helpful, and that’s well appreciated. - Elder, St. George	Physical health, emotional well-being
Education	22	117	I don’t know if – if really people teach anybody to age well. But just by watching, you know, that’s – that’s another thing. That’s another Unangax tradition is you watch and you learn. You didn’t really use words – especially before the Russian contact days. The Unangax people were people of very few words. They talked only when they had to. Especially for – to the – to the young. They were expected to watch and learn and, as they grow, if they felt they were ready to try something, they would be allowed to try it and, you know, learn like that. And still today you’ll hear elders say that, you know, just – words were not wasted. You know? - Elder APIA in ANC	Native way of life, Community engagement, Knowing how to age
Emotional well-being	25	391	But I love it here. I was born and raised here. I like the quietness. I love the clean air and I love the ocean. I love the birds and stuff. And I have my books I, I mean, I read. I would read all of the time. I would read probably about five to six books a week. - Elder, St. George	Native Rural vs urban
Family	25	599	It does because it keeps me on my feet. It keeps me positive and a lot of love from your grandchildren when you see them and how happy you make them. Just watching them grow and how happy they are with you, how much they trust you, how much they wanna be with you. My other grandchildren for my son, when they come to the house, they’re always all excited to come over and spend time with us. So, it makes you feel good about what you’re doing for them and puts you at ease that you’re doing right. So it keeps you calm and stress-free. So, that helps. That helps on a daily basis, and no matter how tired you get, you get that burst of energy always around them, yeah. - Elder APIA in ANC	Emotional well-being, Staying active
History	12	44	1942, I was 13 years old when the Japanese _____ bombed this harbor. I was a kid, so I didn’t mind, care what was going on. I’d see people crying. Me, I didn’t have a feeling about what was going on. They told us to go out – go in a ditch, hide in a cave or someplace, so they took us out. They put us in – it was a big cave. It was cold. Afterwards, they _____ a lot of army there at _____. Then they all came down. Everybody had guns. I wondered what was going on. I didn’t know they had bombed the harbor. So _____ dig a hole in the mountain, not rock. Like, it’s all solid ground, a big hole _____, so we lived inside of that, in the cave. Cause no lights, nothing, one candle. We used one candle to see what were people doing in the cave. Of course, we didn’t wanna play out there. No, they can’t let us. Armies were going, walking back and forth, no lights. We had one candle. That’s all the light we – in daytime, they let us go to our homes. Of course, I didn’t – I had a house _____. Of course, my parents died before the war. My dad died when _____ was running from Seattle down to Nikulski. He hauled supplies from Seattle down along the chain. I never see him anyway, but I barely see my mother though. She gave me away because she was – she knew she was gonna die. – Elder, Unalaska	Family
Housing	11	24	I missed the ocean. I missed the waves. My house is right near the beach. So, on St. George, it’s right down from the church. Always listening to the waves crashing all the time, so the birds flying by. So, it was hard to sleep in quiet. I had a hard time. My daughter was lucky; she had a Christmas gift that she had with her that was aroma therapy, and it had the wave noise to it. So, I put it in my room to sleep. So, that was hard. - Elder APIA in ANC	Rural vs urban, Native way of life, Emotional well-being
Knowing how to age	23	73	When I grew up, it’s because you believe. It’s religion and it’s your culture. It’s your language. It’s your food. [Laughs] It’s your family. You just grow with it. I didn’t see my mother worry so much or stress so much. Less worries, less stress. That’s how I see it because I read on Facebook that all those things affect your body, your system, your liver, your brain.	Family, Physical health, Laughter, Emotional well-being, Native way of life Religion/spirituality, technology
Laughter	17	226	How I became an elder? [Laughs] That’s kind of funny, but I don’t know. I mean how could you become an elder besides growing old every day? [Laughs]	Emotional well-being, Becoming an Elder, Physical health
Native way of life	24	395	That’s how we learned, we learned from other people, from our own experiences. Our own experiences would probably be trial and error but we could learn a lot by paying attention to what other people try to tell us. So, learn to do many different things, not just do one single job. So when I was growing up, I learned to do almost everything that you could do in this town. I learned how to be a health aid, I learned how to be a corporation board member, schoolboard, city council member, I learned how to raise children, I learned how to cook, I learned how to build crab pots, I learned how to fish salmon, I learned how to hunt seals, I learned how to make clothes, I learned how to garden. - Elder, Unalaska	Community engagement, Subsistence, Work, Knowing how to age
Physical health	25	389	Yeah because for the longest time, I denied that I had the rheumatoid arthritis. I would push myself to as far as I could go without complaining, not letting it get the best of me, in other words. But now, it’s a struggle. Especially my granddaughters, we haven’t seen them as much. It’s hard for them not to be able to come and see us. So, just accepting it helps you keep going and just doing what you can with what you got. - Elder, Unalaska	Family, Emotional well-being
Religion, Spirituality	19	103	The day? The first thing I do when I get up and my spouse gets up, or she’s still sleeping, the first thing I do is go in a room – well, first I grab my cup of coffee; an addiction, gotta have my caffeine – and I go in by myself and I spend the first half-hour in private prayer. And that’s the first thing I do each morning, is before I can think of the daily things we all do, what you gotta do, I have to, you got to, you must – all these shoulda/woulda/coulda words that come in our mind – the first thing I have to do is do it with prayer to my God. - Elder, APIA in ANC	Emotional well-being
Role of Elder	16	54	They were too old, especially in the wintertime. They were too old, so they needed help. I was happy I did, grateful for it, because that’s who we learn from. They pass on down things to you and you’ve got to pass it on down to your kids, everything you learned, you know. You could have a college degree and everything, but where you learn is your home. That’s where you first start, from you, your parents, grandparents, elders. You pass that on. - Elder APIA in ANC	Community Engagement, Family, Native way of Life, Emotional well-being
Rural vs urban aging	24	188	I think in general it would be the comparisons. To be able to see those that are still living there that are still productive, and that are still communicating, and still have their families, and comparing with that, I’m doing it here, too. And so, being able to make those comparisons, and be able to just say, “Wow, that’s a blessing they have that,” and to be able to say, “I’m here. It’s a blessing to be here, in this moment.” - Elder APIA ANC	Emotional well-being
Staying active	24	173	And stay involved with even the community public things, go to things that are happening, meetings and big things that are happening in the community. Go to the potlucks, intermingle with people. My mom was shy to go out, we always had to go and get her, and then once she went out to potlucks and dinners and stuff, then she was always so happy at the end. - Elder, Unalaska	Family, Community engagement, Emotional well-being, Advice for youth
Subsistence	20	160	But after the ‘70s, ‘80s and stuff, they started depending on the store more. And they just got in trouble. There’s no going back. And the seals quick kills, they started getting smaller and smaller. Like we killed 200 or so. That’s a lot of meat to put away. But as I’ve grown – as the years moved on, no one was collecting meat any more. So the kill got smaller, from 200 – now they only kill 5. And there’s only a few people that go out there to kill seals any more. And the people are complaining we don’t have any food. - Elder, St. George	Change, Community resources
Technology	16	34	Well, generally, in this day and age as we’ve seen it, media and socially on the networks, I’ve seen changes, and I think they’ve seen changes, too, so. I know the elders who are trying to learn the way it is today, and even this technology, even if they know the minimum technology, they seem to be more into today, and understanding more today. Those that have not had the opportunity to learn of the today-way things are done – again, technology or the way they communicate today – they have found a separation in themselves. - Elder APIA in ANC	Change, Emotional well-being
Transportation	14	48	Years ago, I think it was harder for elders because we had no plane till this year, and when they get sick, more than likely they pass away without good medical help at the time. But we have an airport and we have Medevac access now. So it improved somewhat. If it wasn’t for that, I probably wouldn’t be alive today. - Elder, St. George	Change, Physical health
Work	22	181	Yeah, well, I’ve been pretty much, you know, a jack of all trades. What I needed to do to survive. And I’ve lived in the village and worked for 26 years as a bilingual teacher at the school there in Atka. And then I’ve worked different levels in the school district there in the village and then – but originally I went back as – and when I worked there, when the dentist would come, as a dental assistant, but that was just a once a year contract thing. - Elder APIA in ANC	Native way of life
